# Disturbance of Interaffectivity as a Precursor for Violence in Schizophrenia

**DOI:** 10.1192/j.eurpsy.2025.234

**Published:** 2025-08-26

**Authors:** S. Jerotic

**Affiliations:** 1 Faculty of Medicine, University of Belgrade; 2 Clinic for Psychiatry, University Clinical Centre of Serbia, Belgrade, Serbia

## Abstract

**Abstract:**

Schizophrenia is associated with impaired mentalizing abilities, often conceptualized through Theory of Mind (ToM) paradigms, which highlight deficits in understanding cognitive and affective mental states. Empirical findings suggest that affective ToM impairments reduce the likelihood of violence, while deficits in cognitive ToM may increase its propensity. However, phenomenological approaches challenge the primacy of ToM, suggesting that schizophrenia’s core disturbance lies in the embodied self, specifically in the domain of interaffectivity—the pre-reflective, affective resonance between self and others.

Interaffectivity, rooted in early embodied interactions, forms the foundation of social and emotional connectedness. In schizophrenia, disruptions in this fundamental capacity result in a disconnection from the social environment and a breakdown in shared affective states. This disturbance may contribute to violence by impairing empathic resonance and fostering misinterpretations of social cues.

Phenomenological approaches offer a critical lens for understanding these disturbances, emphasizing the embodied and relational aspects of schizophrenia. By shifting the focus from purely cognitive deficits to fundamental disruptions of interaffectivity, these approaches may provide a roadmap for developing interventions that address the precursors of violence, fostering a multifaceted understanding and management of schizophrenia.

**Disclosure of Interest:**

None Declared

